# Screening tools for sepsis identification in paramedicine and other emergency contexts: a rapid systematic review

**DOI:** 10.1186/s13049-023-01111-y

**Published:** 2023-11-09

**Authors:** Megan De Silva, William Chadwick, Navindhra Naidoo

**Affiliations:** School of Health Sciences: Paramedicine, Western Sydney University, Locked Bag 1797, Penrith, Sydney, NSW 2571 Australia

**Keywords:** Sepsis, Paramedicine, Emergency, Screening tools, qSOFA, SIRS

## Abstract

**Background:**

Sepsis is a life-threatening condition that contributes significantly to protracted hospitalisations globally. The unique positioning of paramedics and other emergency care cadres in emergency contexts enable the prospect of early identification and management of sepsis, however, a standardised screening tool still does not exist in the emergency setting. The objective of this review was to identify and recommend the most clinically ideal sepsis screening tool for emergency contexts such as emergency departments and out-of-hospital emergency contexts.

**Methods:**

A rapid review of five databases (Medline, Embase, the Cochrane Library, CINAHL, and ProQuest Central) was undertaken, with searches performed on February 10, 2022. Covidence software was used by two authors for initial screening, and full text review was undertaken independently by each reviewer, with conflicts resolved by consensus-finding and a mediator. Systematic reviews, meta-analyses, randomised controlled trials, and prospective observational studies were eligible for inclusion. Data extraction used an a priori template and focused on sensitivity and specificity, with ROBINS-I and ROBIS bias assessment tools employed to assess risk of bias in included studies. Study details and key findings were summarised in tables. The a priori review protocol was registered on Open Science Framework (10.17605/OSF.IO/3XQ5T).

**Results:**

The literature search identified 362 results. After review, 18 studies met the inclusion criteria and were included for analysis. There were five systematic reviews, with three including meta-analysis, eleven prospective observational studies, one randomised controlled trial, and one validation study.

**Conclusions:**

The review recognised that a paucity of evidence exists surrounding standardised sepsis screening tools in the emergency context. The use of a sepsis screening tool in the emergency environment may be prudent, however there is currently insufficient evidence to recommend a single screening tool for this context. A combination of the qSOFA and SIRS may be employed to avoid ‘practice paralysis’ in the interim. The authors acknowledge the inherent potential for publication and selection bias within the review due to the inclusion criteria.

## Background

Sepsis is defined as an emergent, life threatening, immunological response to an infectious process that leads to end-stage multi-organ dysfunction and death [1, 2]. The management of sepsis has improved dramatically over the past two decades; however, the importance of early identification cannot be understated, with an increase in mortality of 7.6% for every 6 h of non-identification [3]. Sepsis presents a significant burden of disease accounting for 1.2% of Australian hospitalisations in 2018 [3]. Of these hospitalisations, 12% of patients died, establishing a 10.9 times higher mortality rate than non-sepsis admissions [2]. Sepsis presentations are varied, indiscriminate, and have increased by 27% between 2014 and 2018 [2]. While early differentiation of sepsis from uncomplicated infection is vital, a proverbial ‘gold standard’, validated screening tool still does not exist [4]. This is particularly pertinent within the emergency context, whereby clinicians are well positioned to increase patient outcomes through the early recognition and management of septic patients or those at high risk of sepsis preceding hospital admission. Our premise is that validated, high quality, standardised means of assessing sepsis in the emergency context may consistently achieve early sepsis recognition and intervention [5].

Several screening tools exist to assist in the early identification of sepsis, however, rigorous, high-quality evidence with application to the emergency setting is scarce [6]. Over recent years the quick Sequential Organ Failure Assessment (qSOFA) has gained prominence in some Australian and American jurisdictional ambulance services due to its ease of use in the out of hospital setting and its high sensitivity and specificity for identifying sepsis [1, 4]. However, it remains unclear if this tool is still appropriate for use in the emergency setting, in comparison to other tools such as the Systematic Inflammatory Response Syndrome criteria (SIRS) or Modified Early Warning Score (MEWS). Most sepsis screening tools utilise physiological parameters as a means of assessing potential of deterioration and severity, and thus specific identification of sepsis positive patients is often difficult [7]. The unique positioning of emergency clinicians allows for early and meaningful intervention, thus motivating the need for a validated early recognition screening tool.

To determine the most ideal sepsis screening tool for emergency settings, the authors undertook a rapid systematic review (rapid review) comparing the sensitivity and specificity of the qSOFA application to other reported screening tools. To increase the review’s validity and breadth, the authors widened the emergency setting to include pre-hospital or emergency department (ED) studies that examined these screening tools.

## Methods

The rapid review was undertaken with conformance to the PRISMA 2020 checklist [8] and Joanna Briggs Institute (JBI) evidence summaries [9]. Comprehensive searches of five electronic databases (Medline, Embase, the Cochrane Library, CINAHL, and ProQuest Central) were conducted utilising the PRISMA searching extension on 10 February 2022 to identify potentially relevant studies. Filters were set to include studies published after 2000 in peer-reviewed English-language journals. Due to the time constraints associated with the rapid review methodology, no hand searches of reference lists were conducted. The authors utilised the rapid review methodology to streamline and provide a high-quality, resource-efficient recommendation of the most clinically ideal sepsis screening tool for emergency context, for the key audience of emergency healthcare institutions [10, 11], with respect to the increasing burden of disease secondary to sepsis diagnoses in Australia [2]. This occurred without omission of PRISMA [8] or JBI [9] guidelines, to reduce the risk of bias and to ensure the core systematic principles were upheld [10], whilst expediting essential knowledge synthesis [11]. The full search strategy is listed in Appendix 1. A review protocol was registered on Open Science Framework (10.17605/OSF.IO/3XQ5T) [14].

### Study selection

Both authors (MD and WC) independently reviewed each title and abstract against the pre-defined inclusion criteria using the Covidence software [12]. The pre-defined inclusion criteria were based on the population, intervention, comparison, and outcomes (PICO) acronym, as seen in Table 1.

**Table 1:** Development of PICO

Population	People with suspected or confirmed sepsis in the emergency setting
Intervention	quick Sequential Organ Failure Assessment (qSOFA)
Comparison	Acute sepsis screening tools
Outcomes	Screening tools sensitivity and specificity for the diagnosis of sepsis, and 28- or 30-day mortality

Full text papers that made it through title and abstract screening were independently reviewed by two authors. Systematic reviews and meta-analyses, randomised controlled trials, and prospective observational studies were eligible for inclusion. These coincided with level I and II levels of evidence as defined by the National Health and Medical Research Council (NHMRC) evidence hierarchy to ensure validity, minimise risk of bias, and enhance the overall objective of this paper [13]. Exclusion criteria are listed in Table 2. Studies that met inclusion criteria were put forward for data extraction, and any conflicts were resolved via consensus-finding, with the option of a mediator (NN) where consensus could not be found.

**Table 2:** Exclusion criteria

Wrong setting	Patients not in the emergency setting (defined as pre-hospital, or emergency department)
Wrong populations	Patients without sepsis
Wrong intervention	Not screening tools
Wrong methodology	NHMRC Level III evidence or below
	Studies published before 2000
	Studies published in non-English languages
	Non-peer reviewed academic material
	Irrelevant outcomes (not sensitivity, specificity, or 28- or 30-day mortality)
	No full text paper available
	Studies with high levels of bias as determined by the ROBINS-I or ROBIAS assessment tools

### Quality appraisal

Following the full-text screening, quality appraisal was undertaken by two authors (WC and MD) using the ROBINS-I and ROBIS bias assessment tools [14, 15]. These assessment tools, although not a usual feature of rapid reviews, were utilised due to the heterogeneity of the sources included in the study. The authors note that although using different tools is not ideal, it is vital to appraise potential sources to ensure rigor and validity. Eleven studies were considered to include low levels of bias, and seven were considered to include moderate levels. As a result, no studies were excluded secondary to quality appraisal. Complete quality appraisal findings are listed in Table 3.

**Table 3:** Quality appraisal findings

Author	Risk of bias	
	ROBINS-I Bias Assessment [15]	ROBIS Bias Assessment for Systematic reviews [16]
Abdullah et al. [16]	Moderate	
Azijili et al. [17]	Low	
de Groot et al. [18]	Low	
Feist [19]		Low
Franchini et al. [20]		Low
Graham et al. [25]	Moderate	
Jiang et al. [26]	Low	
Lane et al. [6]	Low	
Loritz et al. [21]	Moderate	
Ortega et al. [22]	Low	
Oduncu et al. [23]	Moderate	
Sabir et al. [7]	Low	
Shirashi et al. [27]	Moderate	
Song et al. [4]	Moderate	
Thodphetch et al. [28]	Moderate	
Waligora et al. [29]		Low
Liu et al. [30]		Low
Yesil et al. [24]	Low	

### Data extraction and synthesis

Data extraction was completed independently by both authors using Covidence software [12]. Data extracted included author/s, year and country of publication, study objective (aim) and methodology, screening tools and patient outcomes. Consensus of data extracted was reached between both authors before a synthesis of evidence was conducted. Patient outcomes were further quantitatively arranged into sensitivity, specificity and 28- and 30-day mortality percentages for each of the screening tools identified through the literature search, during the evidence synthesis.

## Results

### Search and quality appraisal results

**Fig. 1:**
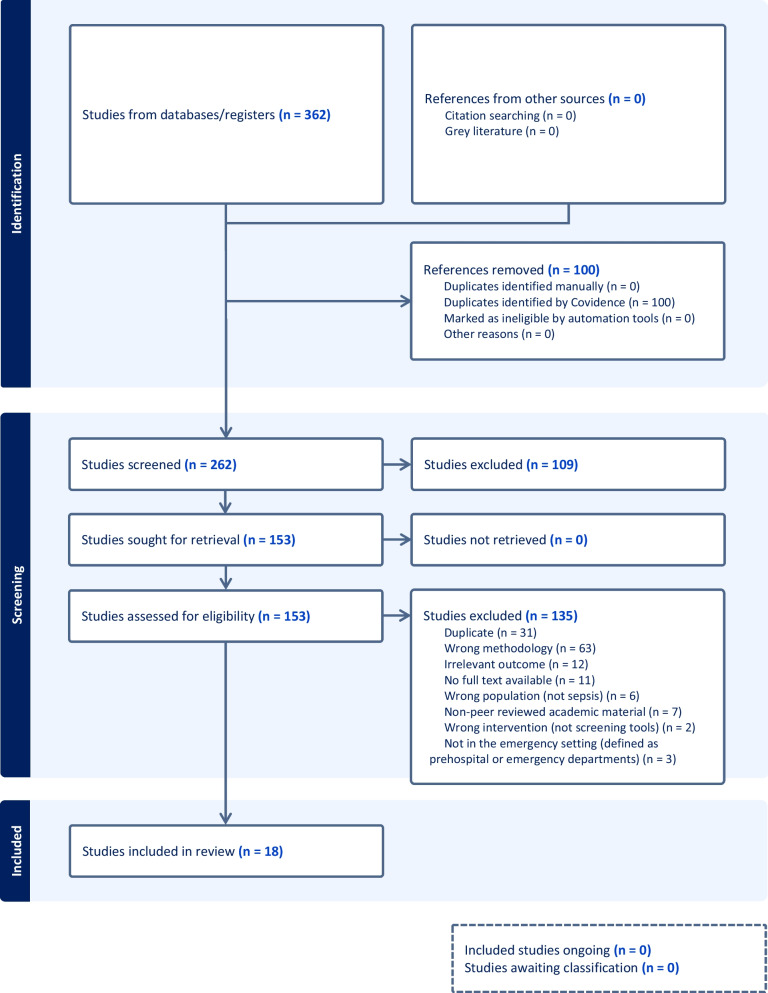
PRISMA diagram

The literature search identified 362 potentially relevant studies. One hundred duplicates were removed via the Covidence software, and the remaining 262 articles were screened on title and abstract, against inclusion criteria. Following the exclusion of 109 irrelevant studies, 152 full text studies were assessed for eligibility. One hundred and thirty-four studies were excluded, including 31 duplicates. Complete exclusion rationale is displayed within the PRISMA diagram (Fig. 1). No studies were excluded secondary to quality appraisal.

### Description of the studies and characteristics of the evidence

Eighteen studies met the inclusion criteria and were included for analysis. Most of the studies were performed in Europe [7, 16–24], and Asia [4, 23–28], with two undertaken in North America [6, 29], and one of an unspecified location [30]. One study was performed in the pre-hospital setting [8], fourteen performed in the emergency department [9, 17–20, 22–30], and three performed in the hospital setting, outside of the intensive care unit [5, 21, 31]. Of these studies, there were five systematic reviews [19, 20, 22, 29, 30], with three including meta-analysis [20, 26, 30], eleven prospective observational studies [7, 16–18, 21, 23, 25, 27, 28, 30], one validation study [6], and one randomised control trial with an ‘all-comer’ design [22]. Each of these studies was deemed to be level I or II evidence by the NHRMC hierarchy [13], and consistent with Grade A recommendations by the American Society of Plastic Surgeons [31]. A summary of included studies is available in Appendix 2.

## Summary of the evidence and the clinical bottom line

Each study evaluated the sensitivity and specificity of qSOFA for the diagnosis of sepsis in the emergency environment, with 28 or 30-day mortality as secondary outcomes. Thirty-one alternative screening tools were identified, with the SIRS criteria and the National Early Warning Score (NEWS) being the most prevalent. Of the included studies the majority indicated that qSOFA produced the highest specificity for emergency diagnosis of sepsis, with low sensitivity. Both Franchini et al. [20] and Jiang et al. [26] proposed that qSOFA may have higher efficacy in identifying patients with suspected infections who are at increased risk of mortality. Of the eighteen studies included in analysis, Lane et al. [6] was the only study performed in the pre-hospital setting, confirming the need for additional research in this field [8]. Lane et al. [6] corroborated the proposals of Franchini et al. [20] and Jiang et al. [26], finding that while NEWS, qSOFA and the Critical Illness Projection score (CIP) have good ability for prehospital sepsis diagnosis, qSOFA’s ease of use may be more advantageous for paramedics [8, 21, 27]. Probable as a clinical bottom line, Graham et al. [25] recommended a combination of qSOFA and SIRS screening tools to improve the prognostic accuracy of 30-day mortality for ED presentations.

## Discussion

Early identification of sepsis in the emergency setting is prudent for early intervention and mortality reduction. With sepsis accounting for extensive emergency presentations, efficacious emergency treatment is essential for reducing incidence and prevalence of ED and intensive care admissions, morbidity, and mortality [2]. To facilitate improved pre-hospital sepsis identification, the Australasian Journal of Paramedicine (now 'Paramedicine') recently supported the inclusion of sepsis screening tools such as qSOFA and SIRS in jurisdictional sepsis screening matrixes [5]. However, the review identified that shortages in high quality pre-hospital research and innovation have prevented validation of a ‘gold standard’ emergency sepsis screening tool [5]. The aim of this rapid review was to determine and recommend an emergency sepsis screening tool for validation, to empower clinicians to successfully identify and initiate sepsis management in the emergency settings. Despite a plethora of previous research, only a minor percentage incorporated the pre-hospital environment outside of, or preceding, the ED. Limited research integrated ambulance services with paramedic data or participation, and none identified a preferred tool for paramedic use, based on efficacy. These findings therefore confirm the unmet research need for further emergency out-of-hospital specific research in sepsis identification, and subsequently, treatment.

Previous literature has queried qSOFA as the preferred screening tool for emergency sepsis presentations [1, 5], therefore, forming the basis of this study’s review. From the evidence analysed within this review, qSOFA demonstrated the highest specificity in differentiating between sepsis and conventional infections without associated organ failure, as per current sepsis definitions [1, 4, 16, 17, 19, 21, 23, 25–28], and most successfully predicted mortality for at risk patients [4, 16, 22, 27, 28]. The authors note that there was a significant range in the specificity and more so, sensitivity in qSOFA results and suggest that is likely due to the heterogeneity of results recorded, particularly considering study designs and settings. The qSOFA low sensitivity was contrasted by SIRS high sensitivity for indicating potentially septic patients [3, 4, 19–21, 23, 26, 27, 29, 32], but with commonly low specificity [4, 23, 24, 26, 27, 29]. According to Feist [19], consequent employment of qSOFA as an emergency sepsis screening tool may reduce ED physician fatigue associated with increased false positives arising from the SIRS criteria. This finding is supported by the Third International Consensus Definitions for Sepsis and Septic Shock (Sepsis-3) [1], and corroborated by Jiang et al. [26], who identified qSOFA as an effective mortality predictor, and Lane et al. [6], concluding qSOFA may be a beneficial sepsis screening tool for paramedics. However, Ortega et al. [22], found that NEWS had the highest combined sensitivity and specificity for predicting sepsis and adverse outcomes for patients within the emergency department, in comparison to qSOFA, based on receiver operator characteristic curves. It is also noted that similar disparities are seen in the in-hospital environment, and a single sepsis identification tool is yet to develop preference. Ortega et al. [22] further described the need for simple and sensitive tools for prompt pre-hospital identification of people at risk of sepsis. With NEWS and qSOFA demonstrating similar performance in identifying patients with sepsis [22], qSOFA requiring only three measures for screening may make it more rapid and favourable for employment in the pre-hospital environment over NEWS [8]. A plethora of evidence exists around sepsis identification in the in-hospital environment and generally suggests that a multi-faceted tiered approach is utilised for sepsis identification and management, including machine learning that includes vital signs and laboratory results aids in the rapid alert of potential sepsis [33]. The in-hospital evidence generally does not favour one specific identification tool [33, 34].

Accordingly, no one screening tool was identified to demonstrate both high sensitivity and specificity for the diagnosis of sepsis [6], rendering the evidence within this review insufficient for recommending a single preferential sepsis screening tool for use within the emergency environment, and more explicitly for paramedicine. As identified by Graham et al. [25], a combination of multiple screening tools employed throughout pre-hospital and ED sepsis presentations may be required to efficiently identify and confirm sepsis diagnosis [25]. Further research which integrates use of qSOFA for suspected sepsis presentations, or SIRS within the emergency environment, followed by qSOFA on ED presentation, is required before a single screening tool can be identified for validation.

### Limitations

The rapid review methodology lends itself to inherent limitations. Firstly, a very specific search was conducted to minimise irrelevant sources, and thus some potential studies may have been excluded simply due to an insufficient search. Exclusion of non-English language papers potentially excluded high quality studies, however due to the nature of a rapid review, the inclusion of said papers was not feasible regarding time and capacity limitations. Exclusion criteria regarding levels of evidence and studies of low quality were intended to increase the strength of the findings; hence a quality appraisal was undertaken. Finally, the review does not include any form of meta-analysis or in-depth quantitative review, in line with the rapid review methodology. The authors declare no conflicts of interest.

## Conclusion

The authors undertook a rapid review to determine the suitability of sepsis screening tools in the emergency setting with regard to sensitivity and specificity of sepsis diagnosis. The review recognised that there is currently a paucity of evidence in the emergency setting and further research is required. When compared to other sepsis screening tools, qSOFA has the highest specificity in differentiation between sepsis and non-septic infections and had the highest prediction rates of mortality. SIRS has a significantly higher sensitivity. The authors propose that the implementation of a sepsis screening tool is prudent in the emergency setting, however further research is required to recommend, validate, and implement a standardised sepsis screening tool. In the interim, qSOFA and SIRS may be used in conjunction to avoid practitioner response paralysis in terms of sepsis risk identification.

## Data Availability

No original data is published, all data is publicly available in referenced results. Search strategies are publicly available in Appendix 1.

## Appendix 1: Full search strategy

**Table:**

**Population**	People with suspected or confirmed sepsis in the emergency setting
Sepsis OR septic?emia* OR py?emia* OR pyohemia* OR bloodstream infection OR blood poisoning	
AND	
ambulance* OR Emergency Medical Technician* OR Air Ambulance* OR emergency medical service* OR paramedic* OR ems OR emt* OR pre?hospital OR first responder* OR emergency service* OR HEMS OR field triage OR triage OR out?of?hospital OR emergency medical technician* OR emergency practitioner* OR emergency department OR accident and emergency OR A&E OR ED OR advanced life support OR emergency rescue* OR emergency resus* OR community support co?ordinator	
**Intervention**	qSOFA OR quick Sequential Organ Failure Assessment
**Comparison**	Acute sepsis screening tools
Acute sepsis screening tool* OR sepsis screening OR sepsis identification OR screening tool*	
OR SIRS criteria Or Systematic Inflammatory Response Syndrome OR GYM score OR MEWS OR Modified Early Warning Score OR MRST OR Robson screening tool OR PRESEP OR Pre?hospital early sepsis detection score OR Sepsis-3 score OR Early Warning Score	
**Outcomes**	Screening tools sensitivity and specificity for the diagnosis of sepsis, and 28- or 30-day mortality
Outcome* OR Effectiv* OR success* OR useful* OR morbidit* OR mortalit* OR clinical effectiv* OR disabilit* OR treatment effectiv*	
**Population AND Intervention AND Comparison AND Outcomes**	

## Appendix 2: Summary of included studies

**Table:**

Author and year of publication	Country/setting	Study methodology/design	Sepsis screening tools analysed	Results/summary			
Abdullah et al. [16]	Denmark Emergency Department	Prospective observational study	qSOFA, SIRS, SOFA		Sensitivity	Specificity	Mortality (28 or 30 days)
				qSOFA	19.6%	92.4%	17.8%
				SIRS	52.5%	51.5%	8.3%
				SOFA	61.4%	67.3%	13.6%
Azijili et al. [17]	Netherlands Emergency Department	Prospective observational study	qSOFA, MEWS, NEWS, SIRS		Sensitivity	Specificity	Mortality (28 or 30 days)
				qSOFA	17.7%	94.2%	
				MEWS	72.6%	54.9%	
				NEWS	75.8%	65.9%	
				SIRS	92.0%	44.6%	
de Groot et al. [18]	Netherlands Emergency Department	Prospective observational study	qSOFA, PIRO, MEDS, MEWS, NEWS		Sensitivity	Specificity	Mortality (28 or 30 days)
				qSOFA	83%	47%	
				PIRO	55%	77%	
				MEDS	81%	62%	
				MEWS	42%	77%	
				NEWS	63%	63%	
Feist [19]	England Emergency Department	Systematic review	qSOFA, SIRS		Sensitivity	Specificity	Mortality (28 or 30 days)
				qSOFA	31%	98%	
				SIRS	67.5%	77%	
Franchini et al. [20]	Italy Emergency Department and Hospital Patients Outside the ICU	Systematic review and meta-analysis	qSOFA, SIRS		Sensitivity	Specificity	Mortality (28 or 30 days)
				qSOFA	51%		5.2%
				SIRS	86%		5.2%
Graham et al. [25]	Hong Kong Emergency Department	Prospective observational study	qSOFA, SIRS, NEWS, qSIRS, NSIRS		Sensitivity	Specificity	Mortality (28 or 30 days)
				qSOFA	11.9%	99.13%	9.5%
				SIRS	44.4%	77.8%	16.7%
				NEWS	35.8%	86.3%	50%
				qSIRS	52.4%	76.4%	12.5%
				NSIRS	36.5%	84.4%	50%
Jiang et al. [26]	China Emergency Department	Meta-analysis	qSOFA, SIRS		Sensitivity	Specificity	Mortality (28 or 30 days)
				qSOFA	42%	88%	
				SIRS	51%	41%	
Lane et al. [6]	Canada Pre-hospital	Validation study	qSOFA, BAS-90-30-90, Borrelli et al., HEWS, MBIS, MEWS, PHANTASi, PITSTOP, PreSAT, PRESEP, PRESS, PSP, qSOFA + ETCO_2_, Robson score, SEPSIS, Sepsis alert, SIRS, SIRS + ETCO_2_, Suffoletto et al		Sensitivity	Specificity	Mortality (28 or 30 days)
				qSOFA	40%	94%	
				BAS-90-30-90	57%	79%	
				Borrelli et al	49%	86%	
				HEWS	85%	41%	
				MBIS	44%	77%	
				MEWS	53%	77%	
				PHANTASi	20%	88%	
				PITSTOP	2%	100%	
				PreSAT	49%	71%	
				PRESEP	49%	76%	
				PRESS	11%	98%	
				PSP	42%	77%	
				qSOFA + ETCO_2_	44%	92%	
				Robson score	75%	54%	
				SEPSIS	26%	94%	
				Sepsis alert	7%	99%	
				SIRS	45%	72%	
				SIRS + ETCO_2_	74%	40%	
				Suffoletto et al	70%	38%	
Loritz et al. [21]	Germany Emergency Department	Prospective observational study	qSOFA, SIRS		Sensitivity	Specificity	Mortality (30 days)
				qSOFA	48.6%	94.3%	57.7%
				SIRS	84.8%	76.7%	69.2%
Ortega et al. [22]	Switzerland Emergency Department	All-comer cohort study	qSOFA, SIRS, NEWS, ESI		Sensitivity	Specificity	Mortality (30 days)
				qSOFA	69.2%	86.5%	3.9%
				SIRS	56.4%	86.4%	2.2%
				NEWS	71.8%	90.2%	1.5%
				ESI	97.4%	32.5%	3.2%
Oduncu et al. [23]	Turkey Emergency Department	Prospective observational study	qSOFA, SIRS, NEWS		Sensitivity	Specificity	Mortality (30 days)
				qSOFA	23%	99.4%	75.8%
				SIRS	77.4%	35.2%	54.2%
				NEWS	58.2%	81.8%	77.2%
Sabir et al. [7]	United Kingdom Emergency Department	Prospective observational study	qSOFA, MEWS, NEWS		Sensitivity	Specificity	Mortality (28 or 30 days)
				qSOFA			57–79%
				MEWS			56–75%
				NEWS			59–88%
Shirashi et al. [27]	Japan Emergency Department	Prospective observational study	qSOFA, SIRS		Sensitivity	Specificity	Mortality (28 or 30 days)
				qSOFA	55%	65%	64%
				SIRS	88%	14%	52%
Song et al. [4]	South Korea Emergency Department	Prospective observational study	qSOFA, SIRS		Sensitivity	Specificity	Mortality (28 or 30 days)
				qSOFA	51%	83%	12.9%
				SIRS	86%	29%	5.8%
Thodphetch et al. [28]	Thailand Emergency Department	Prospective observational study	qSOFA, mNEWS, mSIRS, mSOS		Sensitivity	Specificity	Mortality (30 days)
				qSOFA	47.2%	69.5%	62.7%
				mNEWS	96.4%	17.0%	20.4%
				mSIRS	90.9%	3.4%	15.0%
				mSOS	78.2%	20.3%	28.1%
Waligora et al. [29]	United States Emergency Department	Systematic review	qSOFA, SIRS		Sensitivity	Specificity	Mortality (28 or 30 days)
				qSOFA	58.3%	86–96.7%	
				SIRS	94.5%	45.6–75.2%	
Liu et al. [30]	Unspecified Emergency Department and Hospital Patients Outside the ICU	Systematic review and meta-analysis	qSOFA, SIRS		Sensitivity	Specificity	Mortality (28 or 30 days)
				qSOFA	54%	67%	
				SIRS	72%	71%	
Yesil et al. [24]	Turkey Emergency Department	Prospective observational study	qSOFA, SIRS, qSOFA + SIRS 1, qSOFA + SIRS 2		Sensitivity	Specificity	Mortality (30 days)
				qSOFA	34%	93%	56.1%
				SIRS	81%	31%	27.3%
				qSOFA + SIRS 1	84%	28%	26%
				qSOFA + SIRS 2	31%	96%	56.9%

*qSOFA* quick Sequential Organ Failure Assessment, *SIRS* Systemic Inflammatory Response Syndrome, *SOFA* Sequential Organ Failure Assessment, *MEWS* Modified Early Warning Score, *NEWS* National Early Warning Score, *PIRO* Predisposition Infection Response and Organ Failure, *MEDS* Mortality in ED Sepsis, *qSIRS* qSOFA + SIRS, *NSIRS* NEWS + SIRS, *BAS-90-30-90* Blood Pressure Saturation, *HEWS* Hamilton Early Warning Score, *MBIS* Mecklenburg Bacterial Infection Scale, *PHANTASi* Prehospital Antibiotics Against Sepsis, *PITSTOP* Paramedic Initiated Treatment of Sepsis Targeting Out-of-hospital Patients, *PreSAT* Prehospital Sepsis Assessment Tool, *PRESEP* Prehospital Early Sepsis Detection, *PRESS* Prehospital Severe Sepsis, *PSP* Prehospital Sepsis Project, *qSOFA* + *ETCO*_*2*_ qSOFA + end-tidal carbon dioxide, *SEPSIS* Screening to Enhance Prehospital Identification of Sepsis (SEPSIS), *SIRS* + *ETCO*_*2*_ SIRS + end-tidal carbon dioxide, *ESI* Emergency Severity Index, *mNEWS* modified National Early Warning Score, *mSIRS* modified Systemic Inflammatory Response Syndrome, *mSOS* modified Search Out Severity, *ICU* Intensive Care Unit
